# {*N*,*N*′-Bis­[2-(di­phenyl­phosphan­yl)ethan-1-yl­idene]ethyl­enedi­amine}bromido­(*p*-toluene­sulfonyl­methyl isocyanide)iron(II) tetra­phenyl­borate

**DOI:** 10.1107/S1600536814005467

**Published:** 2014-03-26

**Authors:** Peter E. Sues, Alan J. Lough, Robert H. Morris

**Affiliations:** a Department of Chemistry, University of Toronto, Toronto, Ontario, M5S 3H6, Canada

## Abstract

In the title compound, [FeBr(C_9_H_9_NO_2_S)(C_30_H_30_N_2_P_2_)][B(C_6_H_5_)_4_], the Fe^II^ ion is in a distorted octa­hedral CBrN_2_P_2_ coordination geometry with a P—Fe—P angle of 109.95 (3)°. The relative orientation of the *p*-toluene­sulfonyl­methyl isocyanide ligand is defined by the C—S—C—N torsion angle of 67.1 (2)°. In the crystal, pairs of weak C—H⋯O hydrogen bonds connect the cations into inversion dimers, forming *R*_2_^2^(8) rings.

## Related literature   

For the synthesis, see: Mikhailine *et al.* (2008 ▶). For hydrogen-bond graph-set notation, see: Bernstein *et al.* (1995 ▶).
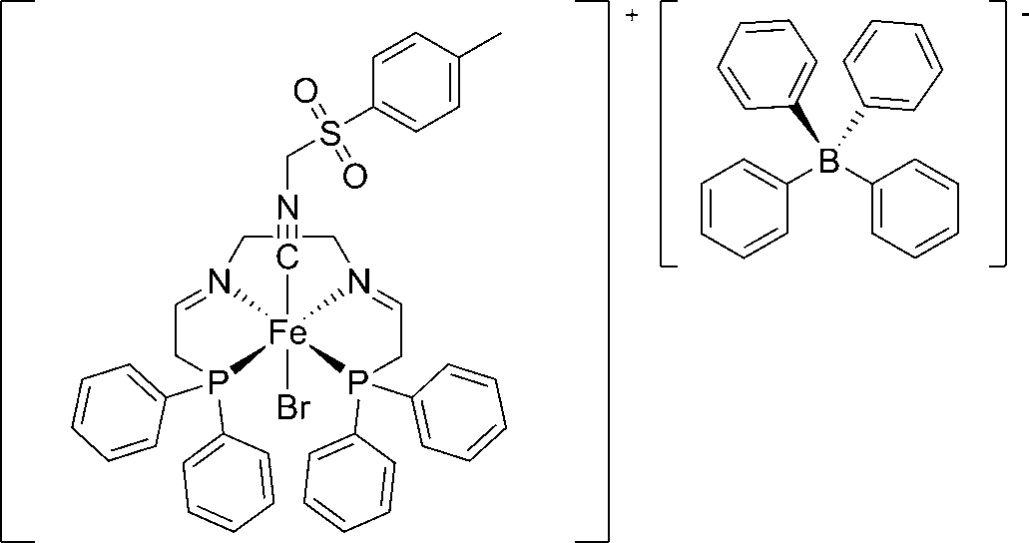


## Experimental   

### 

#### Crystal data  

[FeBr(C_9_H_9_NO_2_S)(C_30_H_30_N_2_P_2_)](C_24_H_20_B)
*M* *_r_* = 1130.70
Triclinic, 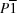

*a* = 13.5738 (16) Å
*b* = 14.1207 (15) Å
*c* = 15.8419 (16) Å
α = 79.847 (4)°
β = 76.873 (5)°
γ = 65.904 (5)°
*V* = 2687.5 (5) Å^3^
*Z* = 2
Cu *K*α radiation
μ = 4.42 mm^−1^
*T* = 147 K
0.05 × 0.03 × 0.02 mm

#### Data collection  

Bruker Kappa APEX DUO CCD diffractometer
Absorption correction: multi-scan (*SADABS*; Bruker, 2012 ▶) *T*_min_ = 0.669, *T*_max_ = 0.753
63879 measured reflections
9108 independent reflections
7945 reflections with *I* > 2σ(*I*)
*R*_int_ = 0.057

#### Refinement  

*R*[*F* ^2^ > 2σ(*F* ^2^)] = 0.034
*wR*(*F* ^2^) = 0.086
*S* = 1.04
9108 reflections
668 parameters
H-atom parameters constrained
Δρ_max_ = 0.48 e Å^−3^
Δρ_min_ = −0.59 e Å^−3^

### 

Data collection: *APEX2* (Bruker, 2012 ▶); cell refinement: *SAINT* (Bruker, 2012 ▶); data reduction: *SAINT*; program(s) used to solve structure: *SHELXS97* (Sheldrick, 2008 ▶); program(s) used to refine structure: *SHELXL2013* (Sheldrick, 2008 ▶); molecular graphics: *PLATON* (Spek, 2009 ▶); software used to prepare material for publication: *SHELXTL* (Sheldrick, 2008 ▶).

## Supplementary Material

Crystal structure: contains datablock(s) I. DOI: 10.1107/S1600536814005467/is5347sup1.cif

Structure factors: contains datablock(s) I. DOI: 10.1107/S1600536814005467/is5347Isup2.hkl

CCDC reference: 990953

Additional supporting information:  crystallographic information; 3D view; checkCIF report

## Figures and Tables

**Table 1:** Hydrogen-bond geometry (Å, °)

*D*—H⋯*A*	*D*—H	H⋯*A*	*D*⋯*A*	*D*—H⋯*A*
C8—H8*A*⋯O2^i^	0.99	2.31	3.099 (3)	136

Symmetry code: (i) 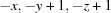.

**Fig. 1.**
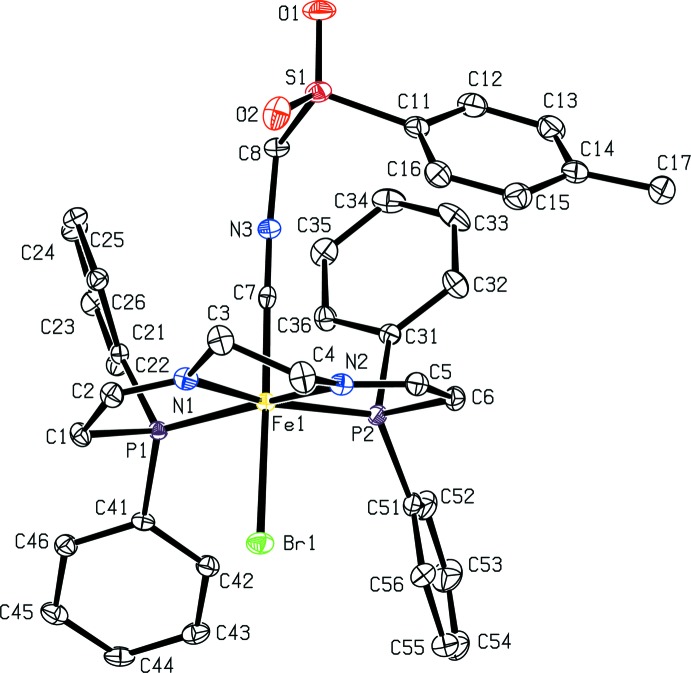
The cation of the title compound showing 30% probability ellipsoids.

**Fig. 2.**
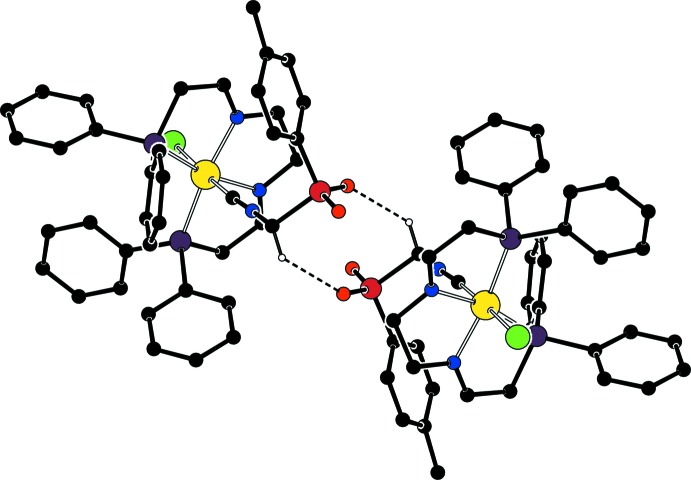
An inversion dimer formed by a pair of weak C—H···O hydrogen bonds (dashed lines) between two cation molecules. Only H atoms involved in hydrogen bonds are shown.

## supplementary crystallographic information

### 1. Comment

The cation of the title compound is shown in Fig. 1. The Fe^II^ ion is in a slightly-distorted octahedral coordination geometry with the P1—Fe1—P2 angle of 109.95 (3)° being the largest deviation from ideal octahedral geometry. The relative orientation of the *para*-toluenesulfonylmethylisocyanide ligand is defined by the C11—S1—C8—N3 torsion angle of 67.1 (2)°. In the crystal, a pair of weak C—H···O hydrogen bonds connect cations into an inversion dimer (Fig. 2) forming an *R*^2^_2_(8) ring (Bernstein *et al.*, 1995).

### 2. Experimental

The synthesis of the precursor [bis(acetonitrile)(*N*,*N*'-bis(2-(diphenylphosphanyl) ethyliedene)ethylenediamine) iron(II) tetraphenylborate] followed a previously published procedure (Mikhailine *et al.*, 2008). Excess KBr (0.2 g, 1.68 mmol) was added to a solution of bis(acetonitrile)(*N*,*N*'-bis(2-(diphenylphosphanyl)ethyliedene)ethylenediamine) iron(II) tetraphenylborate (0.15 g, 0.119 mmol) in acetone (4 ml) in a three-necked round-bottom flask equipped with a magnetic stirring bar, a gas inlet, a reflux condenser and a rubber septum under an inert N_2_ atmosphere. *para*-Toluenesulfonylmethyl isocyanide (0.024 g, 0.119 mmol) in acetone (5 ml) was added with stirring at ambient temperatures. The flask was then placed into an oil bath and the reaction mixture heated to 313 K and stirred at this temperature for 30 min. The reaction mixture was allowed to cool to room temperature, hexanes (25 ml) was added, and the product isolated by filtration. The yellow precipitate was washed with water (2×10 ml), methanol (2×10 ml), and diethyl ether (2×10 ml)(Yield = 0.108 g, 80%). X-ray diffraction quality crystals were grown from the slow diffusion of diethyl ether into a dimethylsulfoxide solution of the title compound.

### 3. Refinement

Hydrogen atoms were placed in calculated positions with C—H = 0.95–0.99 Å and included in the refinement in a riding-model approximation with *U*_iso_(H) = 1.2*U*_eq_(C) or 1.5*U*_eq_(C_methyl_).

### Crystal data

**Table:**

[FeBr(C_9_H_9_NO_2_S)(C_30_H_30_N_2_P_2_)](C_24_H_20_B)	*Z* = 2
*M**_r_* = 1130.70	*F*(000) = 1172
Triclinic, *P*1	*D*_x_ = 1.397 Mg m^−^^3^
*a* = 13.5738 (16) Å	Cu *K*α radiation, λ = 1.54178 Å
*b* = 14.1207 (15) Å	Cell parameters from 9992 reflections
*c* = 15.8419 (16) Å	θ = 3.6–65.8°
α = 79.847 (4)°	µ = 4.42 mm^−^^1^
β = 76.873 (5)°	*T* = 147 K
γ = 65.904 (5)°	Prism, yellow
*V* = 2687.5 (5) Å^3^	0.05 × 0.03 × 0.02 mm

### Data collection

**Table:**

Bruker Kappa APEX DUO CCD diffractometer	7945 reflections with *I* > 2σ(*I*)
Radiation source: Bruker ImuS with multi-layer optics	*R*_int_ = 0.057
φ and ω scans	θ_max_ = 66.4°, θ_min_ = 2.9°
Absorption correction: multi-scan (*SADABS*; Bruker, 2012)	*h* = −15→15
*T*_min_ = 0.669, *T*_max_ = 0.753	*k* = −16→16
63879 measured reflections	*l* = −18→18
9108 independent reflections	

### Refinement

**Table:**

Refinement on *F*^2^	0 restraints
Least-squares matrix: full	Hydrogen site location: inferred from neighbouring sites
*R*[*F*^2^ > 2σ(*F*^2^)] = 0.034	H-atom parameters constrained
*wR*(*F*^2^) = 0.086	*w* = 1/[σ^2^(*F*_o_^2^) + (0.0408*P*)^2^ + 2.0605*P*] where *P* = (*F*_o_^2^ + 2*F*_c_^2^)/3
*S* = 1.04	(Δ/σ)_max_ = 0.001
9108 reflections	Δρ_max_ = 0.48 e Å^−^^3^
668 parameters	Δρ_min_ = −0.59 e Å^−^^3^

### Special details

**Table:**

Geometry. All e.s.d.'s (except the e.s.d. in the dihedral angle between two l.s. planes) are estimated using the full covariance matrix. The cell e.s.d.'s are taken into account individually in the estimation of e.s.d.'s in distances, angles and torsion angles; correlations between e.s.d.'s in cell parameters are only used when they are defined by crystal symmetry. An approximate (isotropic) treatment of cell e.s.d.'s is used for estimating e.s.d.'s involving l.s. planes.

### Fractional atomic coordinates and isotropic or equivalent isotropic displacement parameters (Å^2^)

**Table:**

	*x*	*y*	*z*	*U*_iso_*/*U*_eq_	
Fe1	0.24184 (3)	0.24713 (3)	0.25224 (2)	0.01682 (9)	
Br1	0.33565 (2)	0.07030 (2)	0.20309 (2)	0.02982 (8)	
S1	0.13182 (5)	0.54658 (5)	0.45590 (4)	0.02714 (14)	
P1	0.10168 (5)	0.25748 (4)	0.19371 (4)	0.01708 (13)	
P2	0.34887 (5)	0.31773 (4)	0.15529 (4)	0.01916 (13)	
O1	0.07408 (17)	0.64937 (16)	0.48384 (13)	0.0438 (5)	
O2	0.12548 (16)	0.45826 (16)	0.51375 (12)	0.0380 (5)	
N1	0.16915 (17)	0.18657 (15)	0.35637 (13)	0.0236 (4)	
N2	0.35614 (17)	0.20841 (15)	0.32337 (13)	0.0232 (4)	
N3	0.12910 (16)	0.45064 (15)	0.32730 (13)	0.0233 (4)	
C1	0.0464 (2)	0.17330 (19)	0.27661 (16)	0.0245 (5)	
H1A	−0.0346	0.2058	0.2883	0.029*	
H1B	0.0685	0.1046	0.2550	0.029*	
C2	0.0899 (2)	0.15986 (19)	0.35777 (16)	0.0260 (5)	
H2A	0.0587	0.1314	0.4106	0.031*	
C3	0.2166 (2)	0.1740 (2)	0.43463 (16)	0.0314 (6)	
H3A	0.2019	0.1189	0.4773	0.038*	
H3B	0.1843	0.2400	0.4625	0.038*	
C4	0.3391 (2)	0.1438 (2)	0.40458 (17)	0.0327 (6)	
H4A	0.3717	0.1555	0.4497	0.039*	
H4B	0.3746	0.0691	0.3944	0.039*	
C5	0.4415 (2)	0.22964 (19)	0.30114 (16)	0.0253 (5)	
H5A	0.4926	0.2085	0.3397	0.030*	
C6	0.4618 (2)	0.2870 (2)	0.21575 (16)	0.0255 (5)	
H6A	0.5321	0.2441	0.1816	0.031*	
H6B	0.4672	0.3523	0.2245	0.031*	
C7	0.17161 (18)	0.37228 (18)	0.29535 (14)	0.0187 (5)	
C8	0.0817 (2)	0.54972 (18)	0.35872 (15)	0.0231 (5)	
H8A	0.0010	0.5721	0.3719	0.028*	
H8B	0.0997	0.6010	0.3134	0.028*	
C11	0.2713 (2)	0.5238 (2)	0.42036 (16)	0.0279 (6)	
C12	0.3000 (3)	0.6035 (2)	0.37254 (19)	0.0376 (7)	
H12A	0.2454	0.6678	0.3555	0.045*	
C13	0.4097 (3)	0.5877 (2)	0.3500 (2)	0.0424 (7)	
H13A	0.4301	0.6414	0.3161	0.051*	
C14	0.4909 (2)	0.4949 (2)	0.37594 (18)	0.0378 (7)	
C15	0.4603 (2)	0.4147 (2)	0.42017 (19)	0.0387 (7)	
H15A	0.5151	0.3495	0.4351	0.046*	
C16	0.3508 (2)	0.4283 (2)	0.44297 (18)	0.0330 (6)	
H16A	0.3305	0.3730	0.4736	0.040*	
C17	0.6088 (3)	0.4833 (3)	0.3573 (2)	0.0544 (9)	
H17A	0.6534	0.4164	0.3852	0.082*	
H17B	0.6151	0.5402	0.3803	0.082*	
H17C	0.6347	0.4856	0.2942	0.082*	
C21	−0.01598 (18)	0.38122 (17)	0.19108 (15)	0.0190 (5)	
C22	−0.0468 (2)	0.43710 (18)	0.11316 (16)	0.0228 (5)	
H22A	−0.0102	0.4081	0.0595	0.027*	
C23	−0.1308 (2)	0.5348 (2)	0.11377 (17)	0.0294 (6)	
H23A	−0.1519	0.5721	0.0604	0.035*	
C24	−0.1840 (2)	0.5786 (2)	0.19106 (18)	0.0321 (6)	
H24A	−0.2400	0.6466	0.1907	0.039*	
C25	−0.1556 (2)	0.5232 (2)	0.26921 (17)	0.0285 (6)	
H25A	−0.1933	0.5525	0.3226	0.034*	
C26	−0.07257 (19)	0.42549 (19)	0.26938 (16)	0.0229 (5)	
H26A	−0.0535	0.3878	0.3231	0.028*	
C31	0.2860 (2)	0.46004 (18)	0.13921 (15)	0.0209 (5)	
C32	0.3318 (2)	0.5265 (2)	0.15317 (18)	0.0324 (6)	
H32A	0.4050	0.4991	0.1636	0.039*	
C33	0.2701 (3)	0.6336 (2)	0.15184 (19)	0.0390 (7)	
H33A	0.3015	0.6786	0.1622	0.047*	
C34	0.1652 (3)	0.6745 (2)	0.13582 (18)	0.0359 (7)	
H34A	0.1234	0.7472	0.1368	0.043*	
C35	0.1197 (2)	0.6100 (2)	0.11815 (17)	0.0322 (6)	
H35A	0.0477	0.6381	0.1050	0.039*	
C36	0.1807 (2)	0.50367 (19)	0.12002 (16)	0.0261 (5)	
H36A	0.1496	0.4594	0.1078	0.031*	
C41	0.11374 (19)	0.21067 (17)	0.09031 (15)	0.0199 (5)	
C42	0.2042 (2)	0.20105 (19)	0.02548 (16)	0.0252 (5)	
H42A	0.2626	0.2155	0.0360	0.030*	
C43	0.2101 (2)	0.1703 (2)	−0.05490 (17)	0.0300 (6)	
H43A	0.2718	0.1649	−0.0993	0.036*	
C44	0.1263 (2)	0.14770 (19)	−0.07000 (16)	0.0285 (6)	
H44A	0.1301	0.1273	−0.1251	0.034*	
C45	0.0365 (2)	0.15467 (19)	−0.00490 (17)	0.0296 (6)	
H45A	−0.0202	0.1372	−0.0149	0.036*	
C46	0.0293 (2)	0.18725 (19)	0.07497 (16)	0.0247 (5)	
H46A	−0.0330	0.1935	0.1190	0.030*	
C51	0.42044 (19)	0.2781 (2)	0.04679 (16)	0.0251 (5)	
C52	0.4016 (2)	0.3466 (2)	−0.02799 (17)	0.0352 (6)	
H52A	0.3534	0.4173	−0.0233	0.042*	
C53	0.4532 (3)	0.3119 (3)	−0.1095 (2)	0.0487 (8)	
H53A	0.4401	0.3593	−0.1602	0.058*	
C54	0.5233 (3)	0.2091 (3)	−0.1177 (2)	0.0514 (9)	
H54A	0.5576	0.1858	−0.1737	0.062*	
C55	0.5430 (3)	0.1412 (3)	−0.0444 (2)	0.0477 (8)	
H55A	0.5916	0.0707	−0.0499	0.057*	
C56	0.4925 (2)	0.1746 (2)	0.0379 (2)	0.0359 (6)	
H56A	0.5070	0.1268	0.0882	0.043*	
C61	1.0789 (2)	0.00682 (19)	−0.28860 (19)	0.0302 (6)	
C62	1.0266 (2)	0.0422 (2)	−0.3622 (2)	0.0421 (7)	
H62A	1.0705	0.0330	−0.4185	0.051*	
C63	0.9142 (3)	0.0897 (2)	−0.3554 (3)	0.0537 (9)	
H63A	0.8821	0.1126	−0.4066	0.064*	
C64	0.8484 (3)	0.1038 (2)	−0.2751 (3)	0.0548 (10)	
H64A	0.7711	0.1362	−0.2707	0.066*	
C65	0.8952 (3)	0.0707 (2)	−0.2002 (3)	0.0490 (9)	
H65A	0.8501	0.0802	−0.1444	0.059*	
C66	1.0101 (2)	0.02279 (19)	−0.2078 (2)	0.0358 (7)	
H66A	1.0416	0.0007	−0.1564	0.043*	
C71	1.2732 (2)	−0.09212 (18)	−0.39262 (16)	0.0263 (6)	
C72	1.2376 (2)	−0.1542 (2)	−0.42926 (18)	0.0354 (6)	
H72A	1.1738	−0.1659	−0.3995	0.042*	
C73	1.2918 (3)	−0.1992 (2)	−0.5074 (2)	0.0424 (8)	
H73A	1.2655	−0.2416	−0.5286	0.051*	
C74	1.3831 (3)	−0.1824 (2)	−0.55364 (19)	0.0442 (8)	
H74A	1.4197	−0.2122	−0.6070	0.053*	
C75	1.4201 (2)	−0.1216 (2)	−0.52095 (17)	0.0359 (7)	
H75A	1.4829	−0.1089	−0.5519	0.043*	
C76	1.3656 (2)	−0.07839 (19)	−0.44224 (16)	0.0267 (6)	
H76A	1.3936	−0.0370	−0.4214	0.032*	
C81	1.24591 (18)	−0.14122 (18)	−0.21894 (15)	0.0205 (5)	
C91	1.25498 (19)	0.04773 (18)	−0.29289 (15)	0.0203 (5)	
C82	1.3511 (2)	0.03139 (19)	−0.26384 (16)	0.0234 (5)	
H82A	1.3896	−0.0352	−0.2365	0.028*	
C92	1.25030 (19)	−0.12591 (19)	−0.13495 (16)	0.0237 (5)	
H92A	1.2391	−0.0580	−0.1237	0.028*	
C83	1.3928 (2)	0.10846 (19)	−0.27321 (16)	0.0266 (6)	
H83A	1.4579	0.0944	−0.2519	0.032*	
C93	1.2702 (2)	−0.2053 (2)	−0.06739 (17)	0.0295 (6)	
H93A	1.2731	−0.1911	−0.0117	0.035*	
C84	1.2859 (2)	−0.3048 (2)	−0.08126 (18)	0.0326 (6)	
H84A	1.2992	−0.3595	−0.0355	0.039*	
C94	1.3391 (2)	0.2056 (2)	−0.31375 (17)	0.0297 (6)	
H94A	1.3676	0.2584	−0.3212	0.036*	
C85	1.2820 (2)	−0.3233 (2)	−0.16267 (18)	0.0329 (6)	
H85A	1.2925	−0.3912	−0.1730	0.039*	
C95	1.2440 (2)	0.22536 (19)	−0.34325 (17)	0.0294 (6)	
H95A	1.2069	0.2918	−0.3715	0.035*	
C86	1.2629 (2)	−0.24353 (19)	−0.23000 (17)	0.0258 (5)	
H86A	1.2613	−0.2589	−0.2856	0.031*	
C96	1.2024 (2)	0.14809 (18)	−0.33170 (16)	0.0246 (5)	
H96A	1.1355	0.1640	−0.3509	0.030*	
B1	1.2134 (2)	−0.0454 (2)	−0.29695 (19)	0.0231 (6)	

### Atomic displacement parameters (Å^2^)

**Table:**

	*U*^11^	*U*^22^	*U*^33^	*U*^12^	*U*^13^	*U*^23^
Fe1	0.01778 (19)	0.01577 (18)	0.01614 (19)	−0.00548 (14)	−0.00380 (15)	−0.00086 (14)
Br1	0.03041 (15)	0.02055 (13)	0.03436 (16)	−0.00383 (11)	−0.00677 (11)	−0.00562 (11)
S1	0.0310 (3)	0.0315 (3)	0.0180 (3)	−0.0101 (3)	−0.0016 (2)	−0.0079 (2)
P1	0.0186 (3)	0.0170 (3)	0.0159 (3)	−0.0072 (2)	−0.0022 (2)	−0.0022 (2)
P2	0.0173 (3)	0.0211 (3)	0.0187 (3)	−0.0069 (2)	−0.0024 (2)	−0.0029 (2)
O1	0.0449 (12)	0.0431 (12)	0.0423 (12)	−0.0091 (9)	−0.0027 (9)	−0.0271 (10)
O2	0.0371 (11)	0.0532 (12)	0.0217 (9)	−0.0197 (9)	−0.0052 (8)	0.0078 (9)
N1	0.0295 (11)	0.0226 (10)	0.0184 (10)	−0.0104 (9)	−0.0049 (9)	0.0009 (8)
N2	0.0251 (11)	0.0204 (10)	0.0224 (11)	−0.0057 (8)	−0.0076 (9)	−0.0011 (8)
N3	0.0244 (11)	0.0247 (11)	0.0207 (10)	−0.0089 (9)	−0.0012 (9)	−0.0066 (9)
C1	0.0287 (13)	0.0243 (12)	0.0244 (13)	−0.0158 (11)	−0.0039 (11)	0.0007 (10)
C2	0.0317 (14)	0.0251 (13)	0.0224 (13)	−0.0153 (11)	−0.0029 (11)	0.0038 (10)
C3	0.0398 (16)	0.0411 (15)	0.0176 (13)	−0.0207 (13)	−0.0102 (11)	0.0068 (11)
C4	0.0362 (15)	0.0362 (15)	0.0276 (14)	−0.0154 (12)	−0.0165 (12)	0.0104 (12)
C5	0.0221 (13)	0.0254 (13)	0.0286 (14)	−0.0053 (10)	−0.0112 (11)	−0.0032 (10)
C6	0.0210 (12)	0.0293 (13)	0.0279 (14)	−0.0110 (10)	−0.0048 (10)	−0.0032 (11)
C7	0.0163 (11)	0.0256 (13)	0.0154 (11)	−0.0104 (10)	−0.0039 (9)	0.0022 (10)
C8	0.0275 (13)	0.0204 (12)	0.0192 (12)	−0.0062 (10)	−0.0035 (10)	−0.0040 (10)
C11	0.0328 (15)	0.0324 (14)	0.0219 (13)	−0.0144 (12)	−0.0036 (11)	−0.0081 (11)
C12	0.0468 (18)	0.0333 (15)	0.0345 (16)	−0.0171 (13)	−0.0029 (13)	−0.0090 (12)
C13	0.0517 (19)	0.0412 (17)	0.0417 (17)	−0.0286 (15)	0.0049 (14)	−0.0125 (14)
C14	0.0408 (17)	0.0491 (18)	0.0316 (15)	−0.0226 (14)	0.0016 (13)	−0.0207 (13)
C15	0.0368 (16)	0.0406 (16)	0.0397 (17)	−0.0110 (13)	−0.0113 (13)	−0.0095 (13)
C16	0.0380 (16)	0.0328 (14)	0.0315 (15)	−0.0156 (12)	−0.0103 (12)	−0.0006 (12)
C17	0.0423 (19)	0.071 (2)	0.060 (2)	−0.0307 (17)	0.0093 (16)	−0.0335 (18)
C21	0.0172 (11)	0.0210 (12)	0.0208 (12)	−0.0098 (9)	−0.0015 (9)	−0.0030 (9)
C22	0.0244 (13)	0.0235 (12)	0.0203 (12)	−0.0100 (10)	−0.0027 (10)	−0.0012 (10)
C23	0.0283 (14)	0.0260 (13)	0.0296 (14)	−0.0058 (11)	−0.0095 (11)	0.0024 (11)
C24	0.0235 (14)	0.0257 (13)	0.0405 (16)	−0.0022 (11)	−0.0047 (12)	−0.0058 (12)
C25	0.0230 (13)	0.0301 (13)	0.0295 (14)	−0.0071 (11)	0.0020 (11)	−0.0122 (11)
C26	0.0212 (12)	0.0260 (12)	0.0217 (12)	−0.0087 (10)	−0.0040 (10)	−0.0030 (10)
C31	0.0266 (13)	0.0218 (12)	0.0146 (11)	−0.0122 (10)	0.0000 (10)	−0.0002 (9)
C32	0.0395 (16)	0.0316 (14)	0.0332 (15)	−0.0187 (12)	−0.0150 (12)	0.0027 (11)
C33	0.065 (2)	0.0277 (14)	0.0367 (16)	−0.0268 (14)	−0.0196 (15)	0.0028 (12)
C34	0.0533 (19)	0.0214 (13)	0.0284 (14)	−0.0108 (12)	−0.0083 (13)	0.0015 (11)
C35	0.0331 (15)	0.0266 (14)	0.0310 (15)	−0.0095 (11)	−0.0055 (12)	0.0071 (11)
C36	0.0287 (14)	0.0232 (12)	0.0279 (14)	−0.0134 (11)	−0.0049 (11)	0.0028 (10)
C41	0.0251 (13)	0.0154 (11)	0.0185 (12)	−0.0064 (9)	−0.0047 (10)	−0.0021 (9)
C42	0.0283 (14)	0.0257 (13)	0.0236 (13)	−0.0124 (11)	−0.0019 (11)	−0.0057 (10)
C43	0.0342 (15)	0.0302 (14)	0.0210 (13)	−0.0098 (11)	0.0018 (11)	−0.0054 (11)
C44	0.0383 (15)	0.0234 (13)	0.0204 (13)	−0.0046 (11)	−0.0094 (11)	−0.0060 (10)
C45	0.0341 (15)	0.0263 (13)	0.0315 (15)	−0.0094 (11)	−0.0131 (12)	−0.0065 (11)
C46	0.0240 (13)	0.0263 (13)	0.0244 (13)	−0.0088 (10)	−0.0051 (10)	−0.0047 (10)
C51	0.0179 (12)	0.0358 (14)	0.0254 (13)	−0.0140 (11)	0.0012 (10)	−0.0090 (11)
C52	0.0300 (15)	0.0494 (17)	0.0270 (14)	−0.0177 (13)	−0.0013 (12)	−0.0041 (13)
C53	0.0466 (19)	0.075 (2)	0.0253 (15)	−0.0273 (18)	0.0020 (14)	−0.0092 (15)
C54	0.0410 (18)	0.093 (3)	0.0343 (18)	−0.0385 (19)	0.0131 (14)	−0.0339 (18)
C55	0.0361 (17)	0.055 (2)	0.057 (2)	−0.0209 (15)	0.0097 (15)	−0.0333 (17)
C56	0.0280 (14)	0.0385 (15)	0.0403 (16)	−0.0127 (12)	0.0048 (12)	−0.0154 (13)
C61	0.0250 (14)	0.0177 (12)	0.0492 (17)	−0.0106 (10)	−0.0093 (12)	0.0035 (11)
C62	0.0355 (17)	0.0324 (15)	0.059 (2)	−0.0135 (13)	−0.0213 (15)	0.0132 (14)
C63	0.0362 (18)	0.0337 (17)	0.090 (3)	−0.0112 (14)	−0.0290 (19)	0.0151 (17)
C64	0.0266 (16)	0.0250 (15)	0.111 (3)	−0.0026 (12)	−0.025 (2)	−0.0037 (17)
C65	0.0334 (17)	0.0264 (15)	0.088 (3)	−0.0136 (13)	0.0064 (17)	−0.0249 (16)
C66	0.0235 (14)	0.0204 (13)	0.066 (2)	−0.0084 (11)	−0.0041 (13)	−0.0162 (13)
C71	0.0342 (14)	0.0211 (12)	0.0240 (13)	−0.0086 (11)	−0.0135 (11)	0.0031 (10)
C72	0.0423 (17)	0.0364 (15)	0.0317 (15)	−0.0152 (13)	−0.0152 (13)	−0.0016 (12)
C73	0.054 (2)	0.0375 (16)	0.0398 (17)	−0.0107 (14)	−0.0234 (15)	−0.0112 (13)
C74	0.055 (2)	0.0456 (18)	0.0263 (15)	−0.0088 (15)	−0.0125 (14)	−0.0069 (13)
C75	0.0448 (17)	0.0318 (14)	0.0227 (14)	−0.0081 (13)	−0.0048 (12)	0.0004 (11)
C76	0.0339 (14)	0.0210 (12)	0.0210 (13)	−0.0059 (11)	−0.0087 (11)	0.0021 (10)
C81	0.0132 (11)	0.0214 (12)	0.0238 (12)	−0.0054 (9)	0.0009 (9)	−0.0032 (10)
C91	0.0205 (12)	0.0214 (12)	0.0165 (12)	−0.0073 (10)	0.0014 (9)	−0.0035 (9)
C82	0.0237 (13)	0.0215 (12)	0.0215 (12)	−0.0061 (10)	−0.0017 (10)	−0.0027 (10)
C92	0.0203 (12)	0.0223 (12)	0.0260 (13)	−0.0081 (10)	0.0017 (10)	−0.0041 (10)
C83	0.0232 (13)	0.0297 (13)	0.0270 (13)	−0.0105 (11)	0.0006 (11)	−0.0087 (11)
C93	0.0292 (14)	0.0348 (14)	0.0209 (13)	−0.0125 (11)	0.0006 (11)	0.0007 (11)
C84	0.0335 (15)	0.0282 (14)	0.0288 (14)	−0.0115 (12)	−0.0002 (12)	0.0076 (11)
C94	0.0326 (15)	0.0253 (13)	0.0323 (14)	−0.0154 (11)	0.0046 (12)	−0.0079 (11)
C85	0.0345 (15)	0.0222 (13)	0.0386 (16)	−0.0120 (11)	0.0015 (12)	−0.0023 (11)
C95	0.0324 (15)	0.0203 (12)	0.0316 (14)	−0.0075 (11)	−0.0047 (12)	−0.0002 (11)
C86	0.0252 (13)	0.0239 (13)	0.0268 (13)	−0.0099 (10)	−0.0001 (11)	−0.0033 (10)
C96	0.0256 (13)	0.0238 (12)	0.0232 (13)	−0.0088 (10)	−0.0035 (10)	−0.0018 (10)
B1	0.0215 (14)	0.0222 (14)	0.0260 (15)	−0.0080 (11)	−0.0061 (12)	−0.0013 (11)

### Geometric parameters (Å, º)

**Table:**

Fe1—C7	1.803 (2)	C36—H36A	0.9500
Fe1—N2	1.965 (2)	C41—C42	1.386 (3)
Fe1—N1	1.969 (2)	C41—C46	1.396 (3)
Fe1—P1	2.2420 (7)	C42—C43	1.392 (3)
Fe1—P2	2.2567 (7)	C42—H42A	0.9500
Fe1—Br1	2.4700 (5)	C43—C44	1.378 (4)
S1—O2	1.432 (2)	C43—H43A	0.9500
S1—O1	1.434 (2)	C44—C45	1.388 (4)
S1—C11	1.757 (3)	C44—H44A	0.9500
S1—C8	1.806 (2)	C45—C46	1.391 (3)
P1—C41	1.822 (2)	C45—H45A	0.9500
P1—C21	1.825 (2)	C46—H46A	0.9500
P1—C1	1.852 (2)	C51—C52	1.391 (4)
P2—C51	1.830 (2)	C51—C56	1.398 (4)
P2—C31	1.830 (2)	C52—C53	1.389 (4)
P2—C6	1.853 (2)	C52—H52A	0.9500
N1—C2	1.271 (3)	C53—C54	1.382 (5)
N1—C3	1.475 (3)	C53—H53A	0.9500
N2—C5	1.272 (3)	C54—C55	1.370 (5)
N2—C4	1.471 (3)	C54—H54A	0.9500
N3—C7	1.165 (3)	C55—C56	1.393 (4)
N3—C8	1.409 (3)	C55—H55A	0.9500
C1—C2	1.485 (3)	C56—H56A	0.9500
C1—H1A	0.9900	C61—C66	1.396 (4)
C1—H1B	0.9900	C61—C62	1.412 (4)
C2—H2A	0.9500	C61—B1	1.649 (4)
C3—C4	1.518 (4)	C62—C63	1.381 (4)
C3—H3A	0.9900	C62—H62A	0.9500
C3—H3B	0.9900	C63—C64	1.373 (5)
C4—H4A	0.9900	C63—H63A	0.9500
C4—H4B	0.9900	C64—C65	1.391 (5)
C5—C6	1.479 (4)	C64—H64A	0.9500
C5—H5A	0.9500	C65—C66	1.410 (4)
C6—H6A	0.9900	C65—H65A	0.9500
C6—H6B	0.9900	C66—H66A	0.9500
C8—H8A	0.9900	C71—C76	1.387 (4)
C8—H8B	0.9900	C71—C72	1.411 (4)
C11—C12	1.384 (4)	C71—B1	1.657 (4)
C11—C16	1.388 (4)	C72—C73	1.400 (4)
C12—C13	1.381 (4)	C72—H72A	0.9500
C12—H12A	0.9500	C73—C74	1.376 (5)
C13—C14	1.392 (5)	C73—H73A	0.9500
C13—H13A	0.9500	C74—C75	1.372 (4)
C14—C15	1.385 (4)	C74—H74A	0.9500
C14—C17	1.504 (4)	C75—C76	1.398 (4)
C15—C16	1.387 (4)	C75—H75A	0.9500
C15—H15A	0.9500	C76—H76A	0.9500
C16—H16A	0.9500	C81—C92	1.403 (3)
C17—H17A	0.9800	C81—C86	1.403 (3)
C17—H17B	0.9800	C81—B1	1.650 (4)
C17—H17C	0.9800	C91—C82	1.399 (3)
C21—C22	1.394 (3)	C91—C96	1.401 (3)
C21—C26	1.403 (3)	C91—B1	1.646 (4)
C22—C23	1.385 (3)	C82—C83	1.391 (4)
C22—H22A	0.9500	C82—H82A	0.9500
C23—C24	1.380 (4)	C92—C93	1.391 (4)
C23—H23A	0.9500	C92—H92A	0.9500
C24—C25	1.387 (4)	C83—C94	1.382 (4)
C24—H24A	0.9500	C83—H83A	0.9500
C25—C26	1.380 (3)	C93—C84	1.384 (4)
C25—H25A	0.9500	C93—H93A	0.9500
C26—H26A	0.9500	C84—C85	1.376 (4)
C31—C36	1.388 (4)	C84—H84A	0.9500
C31—C32	1.388 (4)	C94—C95	1.377 (4)
C32—C33	1.398 (4)	C94—H94A	0.9500
C32—H32A	0.9500	C85—C86	1.391 (4)
C33—C34	1.366 (4)	C85—H85A	0.9500
C33—H33A	0.9500	C95—C96	1.389 (4)
C34—C35	1.385 (4)	C95—H95A	0.9500
C34—H34A	0.9500	C86—H86A	0.9500
C35—C36	1.386 (4)	C96—H96A	0.9500
C35—H35A	0.9500		
			
C7—Fe1—N2	90.99 (9)	C34—C33—H33A	119.6
C7—Fe1—N1	88.74 (9)	C32—C33—H33A	119.6
N2—Fe1—N1	82.96 (9)	C33—C34—C35	120.0 (2)
C7—Fe1—P1	95.06 (7)	C33—C34—H34A	120.0
N2—Fe1—P1	164.97 (6)	C35—C34—H34A	120.0
N1—Fe1—P1	83.44 (6)	C34—C35—C36	119.0 (3)
C7—Fe1—P2	87.36 (7)	C34—C35—H35A	120.5
N2—Fe1—P2	84.02 (6)	C36—C35—H35A	120.5
N1—Fe1—P2	166.33 (6)	C35—C36—C31	121.9 (2)
P1—Fe1—P2	109.95 (3)	C35—C36—H36A	119.0
C7—Fe1—Br1	176.19 (7)	C31—C36—H36A	119.0
N2—Fe1—Br1	87.30 (6)	C42—C41—C46	119.4 (2)
N1—Fe1—Br1	87.66 (6)	C42—C41—P1	120.88 (18)
P1—Fe1—Br1	85.80 (2)	C46—C41—P1	119.65 (18)
P2—Fe1—Br1	95.85 (2)	C41—C42—C43	120.4 (2)
O2—S1—O1	119.89 (13)	C41—C42—H42A	119.8
O2—S1—C11	107.42 (12)	C43—C42—H42A	119.8
O1—S1—C11	109.80 (12)	C44—C43—C42	120.0 (2)
O2—S1—C8	108.69 (12)	C44—C43—H43A	120.0
O1—S1—C8	105.36 (12)	C42—C43—H43A	120.0
C11—S1—C8	104.67 (12)	C43—C44—C45	120.1 (2)
C41—P1—C21	102.28 (10)	C43—C44—H44A	119.9
C41—P1—C1	104.54 (11)	C45—C44—H44A	119.9
C21—P1—C1	102.80 (11)	C44—C45—C46	120.1 (2)
C41—P1—Fe1	125.52 (8)	C44—C45—H45A	119.9
C21—P1—Fe1	118.43 (8)	C46—C45—H45A	119.9
C1—P1—Fe1	100.17 (8)	C45—C46—C41	119.9 (2)
C51—P2—C31	104.38 (11)	C45—C46—H46A	120.1
C51—P2—C6	103.41 (11)	C41—C46—H46A	120.1
C31—P2—C6	105.94 (11)	C52—C51—C56	118.6 (2)
C51—P2—Fe1	127.25 (8)	C52—C51—P2	122.0 (2)
C31—P2—Fe1	113.16 (8)	C56—C51—P2	119.3 (2)
C6—P2—Fe1	100.43 (8)	C53—C52—C51	120.2 (3)
C2—N1—C3	122.3 (2)	C53—C52—H52A	119.9
C2—N1—Fe1	124.73 (17)	C51—C52—H52A	119.9
C3—N1—Fe1	112.98 (16)	C54—C53—C52	120.7 (3)
C5—N2—C4	121.3 (2)	C54—C53—H53A	119.6
C5—N2—Fe1	125.02 (17)	C52—C53—H53A	119.6
C4—N2—Fe1	113.48 (16)	C55—C54—C53	119.5 (3)
C7—N3—C8	174.7 (2)	C55—C54—H54A	120.3
C2—C1—P1	108.78 (17)	C53—C54—H54A	120.3
C2—C1—H1A	109.9	C54—C55—C56	120.7 (3)
P1—C1—H1A	109.9	C54—C55—H55A	119.7
C2—C1—H1B	109.9	C56—C55—H55A	119.7
P1—C1—H1B	109.9	C55—C56—C51	120.2 (3)
H1A—C1—H1B	108.3	C55—C56—H56A	119.9
N1—C2—C1	119.9 (2)	C51—C56—H56A	119.9
N1—C2—H2A	120.1	C66—C61—C62	116.1 (3)
C1—C2—H2A	120.1	C66—C61—B1	121.5 (2)
N1—C3—C4	106.7 (2)	C62—C61—B1	122.3 (3)
N1—C3—H3A	110.4	C63—C62—C61	122.4 (3)
C4—C3—H3A	110.4	C63—C62—H62A	118.8
N1—C3—H3B	110.4	C61—C62—H62A	118.8
C4—C3—H3B	110.4	C64—C63—C62	120.3 (3)
H3A—C3—H3B	108.6	C64—C63—H63A	119.8
N2—C4—C3	108.2 (2)	C62—C63—H63A	119.8
N2—C4—H4A	110.1	C63—C64—C65	119.9 (3)
C3—C4—H4A	110.1	C63—C64—H64A	120.0
N2—C4—H4B	110.1	C65—C64—H64A	120.0
C3—C4—H4B	110.1	C64—C65—C66	119.4 (3)
H4A—C4—H4B	108.4	C64—C65—H65A	120.3
N2—C5—C6	120.5 (2)	C66—C65—H65A	120.3
N2—C5—H5A	119.7	C61—C66—C65	121.9 (3)
C6—C5—H5A	119.7	C61—C66—H66A	119.0
C5—C6—P2	109.99 (17)	C65—C66—H66A	119.0
C5—C6—H6A	109.7	C76—C71—C72	113.6 (2)
P2—C6—H6A	109.7	C76—C71—B1	123.6 (2)
C5—C6—H6B	109.7	C72—C71—B1	122.7 (2)
P2—C6—H6B	109.7	C73—C72—C71	123.0 (3)
H6A—C6—H6B	108.2	C73—C72—H72A	118.5
N3—C7—Fe1	176.1 (2)	C71—C72—H72A	118.5
N3—C8—S1	110.59 (17)	C74—C73—C72	120.5 (3)
N3—C8—H8A	109.5	C74—C73—H73A	119.7
S1—C8—H8A	109.5	C72—C73—H73A	119.7
N3—C8—H8B	109.5	C75—C74—C73	118.5 (3)
S1—C8—H8B	109.5	C75—C74—H74A	120.7
H8A—C8—H8B	108.1	C73—C74—H74A	120.7
C12—C11—C16	121.0 (3)	C74—C75—C76	120.2 (3)
C12—C11—S1	118.9 (2)	C74—C75—H75A	119.9
C16—C11—S1	120.0 (2)	C76—C75—H75A	119.9
C13—C12—C11	118.7 (3)	C71—C76—C75	124.2 (3)
C13—C12—H12A	120.7	C71—C76—H76A	117.9
C11—C12—H12A	120.7	C75—C76—H76A	117.9
C12—C13—C14	121.6 (3)	C92—C81—C86	114.8 (2)
C12—C13—H13A	119.2	C92—C81—B1	123.1 (2)
C14—C13—H13A	119.2	C86—C81—B1	122.0 (2)
C15—C14—C13	118.4 (3)	C82—C91—C96	115.2 (2)
C15—C14—C17	121.1 (3)	C82—C91—B1	124.2 (2)
C13—C14—C17	120.4 (3)	C96—C91—B1	119.9 (2)
C14—C15—C16	121.0 (3)	C83—C82—C91	122.8 (2)
C14—C15—H15A	119.5	C83—C82—H82A	118.6
C16—C15—H15A	119.5	C91—C82—H82A	118.6
C15—C16—C11	119.1 (3)	C93—C92—C81	123.2 (2)
C15—C16—H16A	120.4	C93—C92—H92A	118.4
C11—C16—H16A	120.4	C81—C92—H92A	118.4
C14—C17—H17A	109.5	C94—C83—C82	119.7 (2)
C14—C17—H17B	109.5	C94—C83—H83A	120.2
H17A—C17—H17B	109.5	C82—C83—H83A	120.2
C14—C17—H17C	109.5	C84—C93—C92	120.0 (2)
H17A—C17—H17C	109.5	C84—C93—H93A	120.0
H17B—C17—H17C	109.5	C92—C93—H93A	120.0
C22—C21—C26	118.6 (2)	C85—C84—C93	118.7 (2)
C22—C21—P1	122.08 (18)	C85—C84—H84A	120.6
C26—C21—P1	119.14 (18)	C93—C84—H84A	120.6
C23—C22—C21	120.2 (2)	C95—C94—C83	119.6 (2)
C23—C22—H22A	119.9	C95—C94—H94A	120.2
C21—C22—H22A	119.9	C83—C94—H94A	120.2
C24—C23—C22	120.7 (2)	C84—C85—C86	120.8 (2)
C24—C23—H23A	119.7	C84—C85—H85A	119.6
C22—C23—H23A	119.7	C86—C85—H85A	119.6
C23—C24—C25	119.8 (2)	C94—C95—C96	120.0 (2)
C23—C24—H24A	120.1	C94—C95—H95A	120.0
C25—C24—H24A	120.1	C96—C95—H95A	120.0
C26—C25—C24	120.0 (2)	C85—C86—C81	122.5 (2)
C26—C25—H25A	120.0	C85—C86—H86A	118.8
C24—C25—H25A	120.0	C81—C86—H86A	118.8
C25—C26—C21	120.7 (2)	C95—C96—C91	122.7 (2)
C25—C26—H26A	119.6	C95—C96—H96A	118.6
C21—C26—H26A	119.6	C91—C96—H96A	118.6
C36—C31—C32	118.2 (2)	C91—B1—C61	107.87 (19)
C36—C31—P2	117.03 (18)	C91—B1—C81	113.6 (2)
C32—C31—P2	124.48 (19)	C61—B1—C81	108.6 (2)
C31—C32—C33	119.9 (3)	C91—B1—C71	106.2 (2)
C31—C32—H32A	120.0	C61—B1—C71	111.3 (2)
C33—C32—H32A	120.0	C81—B1—C71	109.13 (19)
C34—C33—C32	120.9 (3)		
			
C41—P1—C1—C2	148.07 (17)	C43—C44—C45—C46	1.8 (4)
C21—P1—C1—C2	−105.41 (18)	C44—C45—C46—C41	−1.5 (4)
Fe1—P1—C1—C2	17.05 (18)	C42—C41—C46—C45	−0.1 (4)
C3—N1—C2—C1	−179.3 (2)	P1—C41—C46—C45	177.95 (19)
Fe1—N1—C2—C1	0.3 (3)	C31—P2—C51—C52	−13.4 (2)
P1—C1—C2—N1	−13.1 (3)	C6—P2—C51—C52	−124.1 (2)
C2—N1—C3—C4	143.4 (2)	Fe1—P2—C51—C52	121.4 (2)
Fe1—N1—C3—C4	−36.2 (2)	C31—P2—C51—C56	169.6 (2)
C5—N2—C4—C3	153.1 (2)	C6—P2—C51—C56	59.0 (2)
Fe1—N2—C4—C3	−31.1 (3)	Fe1—P2—C51—C56	−55.5 (2)
N1—C3—C4—N2	42.2 (3)	C56—C51—C52—C53	0.5 (4)
C4—N2—C5—C6	174.7 (2)	P2—C51—C52—C53	−176.5 (2)
Fe1—N2—C5—C6	−0.5 (3)	C51—C52—C53—C54	0.3 (5)
N2—C5—C6—P2	0.1 (3)	C52—C53—C54—C55	−0.8 (5)
C51—P2—C6—C5	−132.30 (18)	C53—C54—C55—C56	0.5 (5)
C31—P2—C6—C5	118.22 (17)	C54—C55—C56—C51	0.2 (4)
Fe1—P2—C6—C5	0.29 (18)	C52—C51—C56—C55	−0.7 (4)
O2—S1—C8—N3	−47.5 (2)	P2—C51—C56—C55	176.3 (2)
O1—S1—C8—N3	−177.15 (18)	C66—C61—C62—C63	−0.2 (4)
C11—S1—C8—N3	67.1 (2)	B1—C61—C62—C63	−177.4 (3)
O2—S1—C11—C12	−171.6 (2)	C61—C62—C63—C64	−0.2 (5)
O1—S1—C11—C12	−39.7 (2)	C62—C63—C64—C65	0.3 (5)
C8—S1—C11—C12	72.9 (2)	C63—C64—C65—C66	0.0 (4)
O2—S1—C11—C16	6.0 (2)	C62—C61—C66—C65	0.5 (4)
O1—S1—C11—C16	137.9 (2)	B1—C61—C66—C65	177.8 (2)
C8—S1—C11—C16	−109.4 (2)	C64—C65—C66—C61	−0.4 (4)
C16—C11—C12—C13	−2.2 (4)	C76—C71—C72—C73	1.2 (4)
S1—C11—C12—C13	175.5 (2)	B1—C71—C72—C73	−175.9 (2)
C11—C12—C13—C14	−1.5 (4)	C71—C72—C73—C74	−1.4 (4)
C12—C13—C14—C15	4.5 (4)	C72—C73—C74—C75	0.7 (4)
C12—C13—C14—C17	−174.6 (3)	C73—C74—C75—C76	0.1 (4)
C13—C14—C15—C16	−3.9 (4)	C72—C71—C76—C75	−0.4 (4)
C17—C14—C15—C16	175.2 (3)	B1—C71—C76—C75	176.7 (2)
C14—C15—C16—C11	0.4 (4)	C74—C75—C76—C71	−0.2 (4)
C12—C11—C16—C15	2.7 (4)	C96—C91—C82—C83	−0.6 (3)
S1—C11—C16—C15	−174.9 (2)	B1—C91—C82—C83	170.1 (2)
C41—P1—C21—C22	−24.7 (2)	C86—C81—C92—C93	0.2 (3)
C1—P1—C21—C22	−132.9 (2)	B1—C81—C92—C93	175.4 (2)
Fe1—P1—C21—C22	117.89 (18)	C91—C82—C83—C94	−0.9 (4)
C41—P1—C21—C26	160.06 (19)	C81—C92—C93—C84	−0.6 (4)
C1—P1—C21—C26	51.8 (2)	C92—C93—C84—C85	0.4 (4)
Fe1—P1—C21—C26	−57.4 (2)	C82—C83—C94—C95	1.0 (4)
C26—C21—C22—C23	0.8 (4)	C93—C84—C85—C86	0.2 (4)
P1—C21—C22—C23	−174.45 (19)	C83—C94—C95—C96	0.4 (4)
C21—C22—C23—C24	0.7 (4)	C84—C85—C86—C81	−0.5 (4)
C22—C23—C24—C25	−1.9 (4)	C92—C81—C86—C85	0.3 (4)
C23—C24—C25—C26	1.5 (4)	B1—C81—C86—C85	−174.9 (2)
C24—C25—C26—C21	0.1 (4)	C94—C95—C96—C91	−1.9 (4)
C22—C21—C26—C25	−1.3 (4)	C82—C91—C96—C95	1.9 (3)
P1—C21—C26—C25	174.16 (19)	B1—C91—C96—C95	−169.1 (2)
C51—P2—C31—C36	90.5 (2)	C82—C91—B1—C61	153.5 (2)
C6—P2—C31—C36	−160.71 (19)	C96—C91—B1—C61	−36.3 (3)
Fe1—P2—C31—C36	−51.6 (2)	C82—C91—B1—C81	33.0 (3)
C51—P2—C31—C32	−96.1 (2)	C96—C91—B1—C81	−156.8 (2)
C6—P2—C31—C32	12.7 (2)	C82—C91—B1—C71	−87.0 (3)
Fe1—P2—C31—C32	121.8 (2)	C96—C91—B1—C71	83.2 (3)
C36—C31—C32—C33	3.1 (4)	C66—C61—B1—C91	−83.2 (3)
P2—C31—C32—C33	−170.3 (2)	C62—C61—B1—C91	93.9 (3)
C31—C32—C33—C34	−0.9 (4)	C66—C61—B1—C81	40.4 (3)
C32—C33—C34—C35	−1.8 (4)	C62—C61—B1—C81	−142.5 (2)
C33—C34—C35—C36	2.2 (4)	C66—C61—B1—C71	160.6 (2)
C34—C35—C36—C31	0.1 (4)	C62—C61—B1—C71	−22.3 (3)
C32—C31—C36—C35	−2.7 (4)	C92—C81—B1—C91	33.7 (3)
P2—C31—C36—C35	171.1 (2)	C86—C81—B1—C91	−151.5 (2)
C21—P1—C41—C42	114.2 (2)	C92—C81—B1—C61	−86.4 (3)
C1—P1—C41—C42	−138.9 (2)	C86—C81—B1—C61	88.5 (3)
Fe1—P1—C41—C42	−24.7 (2)	C92—C81—B1—C71	152.1 (2)
C21—P1—C41—C46	−63.8 (2)	C86—C81—B1—C71	−33.1 (3)
C1—P1—C41—C46	43.1 (2)	C76—C71—B1—C91	19.4 (3)
Fe1—P1—C41—C46	157.29 (15)	C72—C71—B1—C91	−163.7 (2)
C46—C41—C42—C43	1.3 (4)	C76—C71—B1—C61	136.6 (2)
P1—C41—C42—C43	−176.71 (19)	C72—C71—B1—C61	−46.5 (3)
C41—C42—C43—C44	−1.0 (4)	C76—C71—B1—C81	−103.5 (3)
C42—C43—C44—C45	−0.6 (4)	C72—C71—B1—C81	73.4 (3)

### Hydrogen-bond geometry (Å, º)

**Table:**

*D*—H···*A*	*D*—H	H···*A*	*D*···*A*	*D*—H···*A*
C8—H8*A*···O2^i^	0.99	2.31	3.099 (3)	136

Symmetry code: (i) −*x*, −*y*+1, −*z*+1.
